# Screening behaviours, demographics, and stage at diagnosis in the publicly funded Ontario Breast Screening Program

**DOI:** 10.1007/s10549-022-06848-1

**Published:** 2023-02-17

**Authors:** Nicholas Gold, Rebecca A. G. Christensen, Jasleen Arneja, Arian Aminoleslami, Geoffrey M. Anderson, Jennifer D. Brooks

**Affiliations:** 1 Dalla Lana School of Public Health, University of Toronto, 155 College St. HSB 676, Toronto, ON M5T 3M7 Canada; 2 Institute of Health Policy, Management and Evaluation, University of Toronto, Toronto, ON Canada

**Keywords:** Breast cancer, Screening, Diagnosis, Stage, Ontario

## Abstract

**Purpose:**

The Ontario Breast Screening Program (OBSP) offers free screening mammograms every 2 years, to women aged 50–74. Study objectives were to determine demographic characteristics associated with the adherence to OBSP and if women screened in the OBSP have a lower stage at diagnosis than non-screened eligible women.

**Methods:**

We used the Ontario cancer registry (OCR) to identify 48,927 women, aged 51–74 years, diagnosed with breast cancer between 2010 and 2017. These women were assigned as having undergone adherent screening (*N* = 26,108), non-adherent screening (*N* = 6546) or not-screened (*N* = 16,273) in the OBSP. We used multinomial logistic regression to investigate the demographic characteristics associated with screening behaviour, as well as the association between screening status and stage at diagnosis.

**Results:**

Among women with breast cancer, those living in rural areas (versus the largest urban areas) had a lower odds of not being screened (odds ratio [OR] 0.73, 95% confidence interval [CI] 0.68, 0.78). Women in low-income (versus high-income) communities were more likely not to be screened (OR 1.42, 95% CI 1.33, 1.51). When stratified, the association between income and screening status only held in urban areas. Non-screened women were more likely to be diagnosed with stage II (OR 1.91, 95% CI 1.82, 2.01), III (OR 2.96, 95% CI 2.76, 3.17), or IV (OR 8.96, 95% CI 7.94, 10.12) disease compared to stage I and were less likely to be diagnosed with ductal carcinoma in situ (DCIS) (OR 0.91, 95% CI 0.84–0.98).

**Conclusions:**

This study suggests that targeting OBSP recruitment efforts to lower income urban communities could increase screening rates. OBSP adherent women were more likely to be diagnosed with earlier stage disease, supporting the value of this initiative and those like it.

## Introduction

Breast cancer is the most commonly diagnosed malignancy in women worldwide, with one in eight women in Canada expected to develop the disease in their lifetime [1]. In the province of Ontario Canada, the Ontario Breast Screening Program (OBSP) provides breast cancer screening with mammography every 2 years to eligible women in the population (ages 50–74 years). Along with these age criteria, to be eligible, women must have no new breast cancer symptoms, personal history of breast cancer, breast implants, or history of mastectomy [2]. Annual screening is available for women who have a family history of breast or ovarian cancer, extremely dense breasts, or a history of high-risk lesions [2, 3]. In the year 2018, approximately 77% of all women eligible for breast cancer screening within and outside of the OBSP received a screening mammogram within the previous 30 months [3]. While it is good that nearly two-thirds of women in Ontario had received at least one mammogram during this interval, there is still a substantial proportion of screening-eligible women who may be under-screened or not screened at all. This is concerning given the evidence that breast cancer screening leads to the diagnosis of early-stage disease, [4, 5] which in turn is associated with lower breast cancer mortality [6].

Those who undergo breast cancer screening are known to differ from those that do not, based on some key demographic characteristics. For example, it has been shown that individuals living in a rural community [7] or having low-income socio-economic status [8] are less likely to participate in breast cancer screening in the United States and the Netherlands, respectively. Understanding the characteristics of individuals who do not get screened is essential to inform policies seeking to increase participation and adherence in breast screening programmes. This will ensure equitable access to, and sharing of, the benefits of such programmes.

Accordingly, this study had two main objectives. The first was to identify the characteristics associated with participation in the OBSP and the second is to determine if OBSP screening is indeed associated with a lower stage at diagnosis.

## Methods

### Study population

**Fig. 1:**
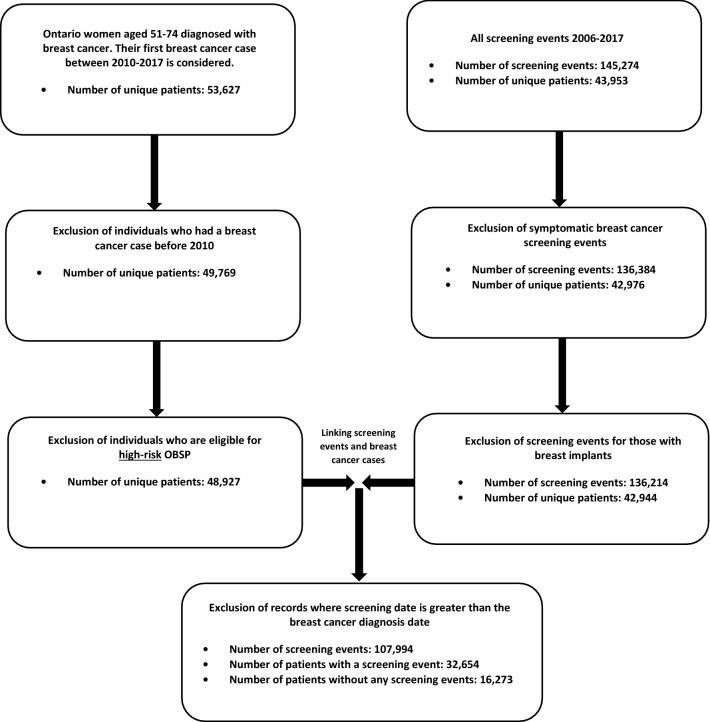
Overview of cohort definition and exclusions for women age 51–74 years diagnosed with breast cancer in the province of Ontario (2010–2017)

The retrospective cohort under investigation was identified through the Ontario Cancer Registry (OCR) and includes records for all women ages 51–74 years in Ontario diagnosed with a first primary breast cancer (invasive or ductal carcinoma in situ [DCIS]) between 2010 and 2017 (*N* = 53,627) (Fig. 1). Information on breast cancer screening was obtained from OBSP records and included 145,274 screening records spanning from 2006 to 2017. These records represented singular screening events from 43,953 unique patients (Fig. 1).

For women with a second primary breast cancer between 2010 and 2017 (*N* = 3,304), only information on their first breast cancer diagnosis in that time-period was included. An individual woman in the sample could have multiple screening events through routine participation in the OBSP. Symptomatic (diagnostic) breast cancer screening events (*N* = 8890) and any screening event for women with breast implants (because screening recommendations in the OBSP differ for women with breast implants, *N* = 170) were excluded. Women in the High Risk OBSP or those who were eligible for the High Risk OBSP (*N* = 842) were excluded, because these individuals follow different screening guidelines [3].

### Screening behaviour

Adherent screeners were defined as those who had one OBSP mammogram at least once every 3 years for those eligible for biennial screening or once every 2 years for those on annual recall screening, starting at age 50 years. We provided women with a grace period of approximately 1 year for both annual and biennial screening to allow for variability due to scheduling. Conversely, women who had been diagnosed with breast cancer but had no record of screening within the OBSP were defined as non-screening individuals. Women who had at least one OBSP screen, but not according to OBSP guidelines, were classified as non-adherent screeners. Because the OBSP starts screening women at age 50 years, women diagnosed prior to age 51 years were not eligible for the current study so we could accurately determine their screening status (i.e. women diagnosed with breast cancer at age 51 years were classified as adherent screeners if screened at age 50 years, and a non-screener if not). After the application of all exclusion criteria, the final cohort included 48,927 individual women (91.2%) (Fig. 1).

### Cancer stage and tumour characteristics

Data based on TNM stage guidelines were used to define cancer stage for this analysis. This classification guideline uses tumour size (T), number of surrounding lymph nodes with cancer (N), and cancer metastasis (M) to determine the stage of cancer at diagnosis. Within this dataset, stage zero cancers included any non-staged forms of breast cancer such as Paget’s disease or Phyllodes tumours [9, 10]. DCIS was classified in its own category. Missing data for cancer stage were classified as unknown cancer stage (*N* = 3565, 7.3%). Data for both estrogen (ER) and progesterone (PR) receptor status and human epidermal growth factor receptor 2 (HER2) status were also obtained from the OCR. The outcomes for these three hormone receptors were classified as positive, negative, or unknown (includes borderline status). If a woman was diagnosed with multi-focal or bilateral disease, characteristics of the highest stage tumour were used.

### Patient characteristics

Data on an individual’s community size, urban, or rural setting and income were obtained from Ontario Health (Cancer Care Ontario). Statistics Canada’s definition of census metropolitan area (CMA) was used to categorize the residential community size [11]. A CMA is defined as urban areas of differing population size from under 100,000 people, 100,000–499,999, 500,000–1,499,999, or over 1,500,000. Individuals who lived outside of CMAs were defined as living in a rural community [11]. Before tax, neighbourhood income quintile was categorized as a three-category variable combining the three middle-income quintiles (middle, lower-middle, upper-middle) into a single middle-income level, while the lowest and the highest income quintiles were maintained as is [12]. All instances where community size or income were missing were coded as ‘Unknown’ (*N* = 743, 1.5%).

### Statistical analyses

Standardized differences (SD) were used to compare patient characteristics (e.g. residential community size, neighbourhood income, age, and presence of a prior non-breast cancer (yes/no)), and tumour characteristics such as cancer stage, ER, PR, and HER2 receptor status between non-screeners, non-adherent screeners, and adherent screeners. A standardized difference greater than or equal to 0.1 was used to represent a meaningful difference between groups [13].

Multivariable adjusted multinomial logistic regression was used to estimate odds ratios (OR) and 95% confidence intervals (CI) for the association between age, neighbourhood income, residential community size, and prior non-breast cancer with screening behaviour (non-screener or non-adherent screener versus adherent screener) in a mutually adjusted model. A second model was run exploring the potential interaction between community size and income quintile using a product term. This led to models being stratified by community size. Multinomial logistic regression (generalized logit) was used to examine the association between screening behaviour (adherent screener versus non-adherent screener or non-screener) and cancer stage at diagnosis (stage I–IV, DCIS, and non-staged cancer). Age, prior non-breast cancer, and residential community size were all treated as confounders for this analysis.

All tests were two-sided, and a p-value of less than 0.05 was considered statistically significant. Data cleaning and standardized differences were performed in R, and all model creation and diagnostics were performed in SAS Studio Version 9.4.

## Results

The median age at breast cancer diagnosis was 63 years, with those who were screened at least once being slightly older than those who were not screened (SD = 0.23). Over a third of the cohort (38.4%) resided in a metropolitan area with a population of more than 1,500,000 people, while 12.1% of the cohort lived in a rural setting (Table 1). Most women also fell within the middle-income category (59.2%), with no difference in screening behaviours observed (SDs < 0.1) (Table 1).

**Table 1:** Demographic variables and breast cancer characteristics stratified by screening behaviour for women ages 51–74 years diagnosed with a first breast cancer in Ontario between 2010 and 2017

	Overall	Adherent screening	Non-adherent screening	Non-screening	Standardized difference^f^
	(*N* = 48,927)	(*N* = 26,108)	(*N* = 6,546)	(*N* = 16,273)	
Age^a^					
Mean (SD)	62.4 (6.7)	62.4 (6.7)	64.0 (5.6)	61.7 (7.1)	**0.23**
Median (IQR)	63 (57–68)	63 (57–68)	64 (59–69)	62 (55–68)	
Neighbourhood income					
Highest	10,459 (21.4%)	5802 (22.2%)	1367 (20.9%)	3290 (20.2%)	0.03
Middle^b^	28,944 (59.2%)	15,740 (60.3%)	3789 (57.9%)	9415 (57.9%)	0.03
Lowest	8781 (18.0%)	4226 (16.2%)	1265 (19.3%)	3290 (20.2%)	0.07
Unknown	743 (1.5%)	340 (1.3%)	125 (1.9%)	278 (1.7%)	0.03
Community size^c^					
Urban population < 100,000	4437 (9.1%)	2447 (9.4%)	557 (8.5%)	1433 (8.8%)	0.02
Urban population 100,000–499,999	10,998 (22.5%)	6520 (25.0%)	1561 (23.9%)	2917 (17.9%)	**0.11**
Urban population 500,000–1,499,999	8046 (16.4%)	4151 (15.9%)	971 (14.8%)	2924 (18.0%)	0.06
Urban Population > 1,500,000	18806 (38.4%)	9302 (35.6%)	2581 (39.4%)	6923 (42.5%)	0.09
Rural area	5897 (12.1%)	3348 (12.8%)	751 (11.5%)	1798 (11.1%)	0.04
Unknown	743 (1.5%)	340 (1.3%)	125 (1.9%)	278 (1.7%)	0.03
Prior non-breast cancer					
No	45,098 (92.2%)	24,058 (92.2%)	5991 (91.5%)	15,049 (92.5%)	0.02^g^
Yes	3829 (7.8%)	2050 (7.9%)	555 (8.5%)	1244 (7.5%)	
Cancer stage					
I	19,587 (40.0%)	12,016 (46.0%)	2705 (41.3%)	4866 (29.9%)	**0.22**
II	14,163 (29.0%)	6830 (26.2%)	1966 (30.0%)	5367 (33.0%)	0.10
III	4726 (9.7%)	1875 (7.2%)	555 (8.5%)	2296 (14.1%)	**0.15**
IV	1851 (3.8%)	365 (1.4%)	190 (2.9%)	1296 (8.0%)	**0.21**
DCIS	4852 (9.9%)	3053 (11.7%)	641 (9.8%)	1158 (7.1%)	**0.11**
Non-Staged^d^	183 (0.4%)	97 (0.4%)	30 (0.5%)	56 (0.3%)	0.01
Unknown	3565 (7.3%)	1872 (7.2%)	459 (7.0%)	1234 (7.6%)	0.01
Estrogen receptor					
Negative	5904 (12.1%)	2922 (11.2%)	744 (11.4%)	2238 (13.8%)	0.05
Positive	32,691 (66.8%)	17,397 (66.6%)	4494 (68.7%)	10,800 (66.4%)	0.03
Unknown^e^	10,332 (21.1%)	5789 (22.2%)	1308 (20.0%)	3235 (19.9%)	0.04
Progesterone receptor					
Negative	9824 (20.1%)	4895 (18.8%)	1316 (20.1%)	3613 (22.2%)	0.06
Positive	28,711 (58.7%)	15,389 (58.9%)	3912 (59.8%)	9410 (57.8%)	0.03
Unknown^e^	10,392 (21.2%)	5824 (22.3%)	1318 (20.1%)	3250 (20.0%)	0.04
HER2 status					
Negative	24,669 (50.4%)	13,254 (50.8%)	3972 (60.7%)	7443 (45.7%)	**0.20**
Positive	4262 (8.7%)	2059 (7.9%)	653 (10.0%)	1550 (9.5%)	0.05
Unknown^e^	19,996 (40.9%)	10,795 (41.4%)	1921 (29.4%)	7280 (44.7%)	**0.21**

*HER2* human epidermal growth factor receptor 2, *DCIS* ductal carcinoma in situ

^a^Age for this cohort was restricted to participants between the age of 50 to 74 to reflect those eligible for the Ontario breast cancer screening programme average risk eligibility group

^b^The middle-income classification represents a combination of all individuals who fell within the middle three quintiles of the neighbourhood income distribution named lower-middle, middle, upper-middle-income

^c^Community size is differentiated by population size. Urban area is defined as being part of a population centre, while rural areas are areas surrounding urban areas without a population centre

^d^Non-staged breast cancer represents invasive stage zero forms of breast cancer such as Paget's disease, Phyllodes tumours, and angiosarcoma of the breast

^e^Unknown receptor status of breast cancer tumours represent both cases that had no data on receptor status and tumours in which results were borderline and unable to be classified

^f^Differences above 0.1 (in bold font) represent meaningfully differences in distributions for a variable level. One standardized difference was calculated by first calculating a unique standardized difference for all possible pairwise. Standardized differences less than 0 were multiplied by -1, and then the average of the three standardized differences was calculated

^g^Since prior cancer is a binary variable, only one standardized difference was generated to show comparisons between the levels, since no ‘dummy’ variables needed to be coded

Overall, stage I cancers were the most (40.0%, *N* = 19,587) and stage IV, the least (3.8%, *n* = 1,851) common stage at diagnosis in the cohort (Table 1). Of all the women diagnosed with breast cancer between 2010 and 2017, 53.4% were classified as being adherent screeners. In unadjusted analyses, the relative fraction of stage I cancers was larger among non-adherent and adherent screeners versus non-screeners (SD = 0.22). Correspondingly, non-screeners had a higher proportion of stage III (SD = 0.15) and IV (SD = 0.21) cancers. DCIS was more commonly diagnosed in adherent screeners compared to non-adherent and non-screening individuals (SD = 0.11).

The majority of cancers with available information on receptor status (ER: 78.9%, PR: 78.8%, HER2: 59.1%) were ER-positive (84.7%), PR-positive (74.5%), and HER2-negative (85.3%). A larger proportion of non-adherent screening women were HER2-negative (SD = 0.20) and a smaller proportion had unknown HER2 receptor status (SD = 0.21), but there was a similar proportion of women with HER2-positive cancers regardless of compliance (SD < 0.1) (Table 1).

### Patient characteristics and screening behaviours

The odds of being a non-screener decreased with every 5-year increase in age (OR 0.93, 95% CI 0.92, 0.95) (Table 2). Compared to those living in an urban area of over 1,500,000 people, the odds of being a non-screener were significantly lower for those living in smaller urban areas, including communities with a population under 100,000 (OR 0.79, 95% CI 0.74, 0.85), between 100,000 and 499,999 people (OR 0.61, 95% CI 0.57, 0.64), and for those living in a rural area (OR 0.73, 95% CI 0.68, 0.78). An association between neighbourhood income and screening status was also observed. Specifically, the odds of being a non-screener compared to an adherent screener were significantly higher for those in the lowest and middle-income categories compared to the highest (OR 1.42, 95% CI 1.33, 1.51 and OR 1.08, 95% CI 1.03, 1.13, respectively).

**Table 2:** Multinomial logistic regression measuring the association between age, community size, neighbourhood income, and prior non-breast cancer with the screening behaviours of Ontario women diagnosed with breast cancer from 2010 to 2017 (*n* = 48,927)

Adjusted risk factor^a^	Non-adherent^b^	Non-screening^b^
	OR (95% CI)	OR (95% CI)
Age^c^	1.19 (1.17, 1.22)	0.93 (0.92, 0.95)
Community size (Compared to urban area 1,500,000 + people)^d^		
Urban 500,000–1,499,999	0.83 (0.77, 0.90)	0.96 (0.90, 1.00)
Urban 100,000–499,999	0.84 (0.78, 0.90)	0.61 (0.57, 0.64)
Urban < 100,000	0.79 (0.71, 0.87)	0.79 (0.74, 0.85)
Rural	0.78 (0.72, 0.86)	0.73 (0.68, 0.78)
Unknown	1.34 (1.08, 1.66)	1.25 (1.06, 1.47)
Neighbourhood income^e^		
Lowest vs. Highest	1.25 (1.15, 1.37)	1.42 (1.33, 1.51)
Middle vs. Highest	1.02 (0.95, 1.09)	1.08 (1.03, 1.13)
Middle vs. Lowest	0.81 (0.76, 0.87)	0.76 (0.72, 0.80)
Prior non-breast cancer^f^		
Yes vs. No	1.02 (0.92, 1.13)	0.98 (0.91, 1.06)

*OR* odds ratio, *CI* confidence interval

^a^In this model there is no focal exposure. The relationships of covariates with screening behaviours are explored

^b^Screening behaviour is considered the outcome of interest for this analysis. Adherent screening behaviour is considered the reference level

^c^Age is a continuous variable increasing in units of 5

^d^Urban and rural community size are a categorical variable. Rural areas are defined as surrounding urban areas that have no population centre of their own. Urban areas with over 1,500,000 people act as the reference level for this variable. No estimate is provided for the ‘Unknown’ group due to the small number of individuals in this category leading to unstable estimates (see Table 1)

^e^Neighbourhood income is a categorical variable with three levels. Highest represents the wealthiest neighbourhood income quintile. Middle represents the combined middle three income quintiles. Lowest represents the poorest neighbourhood income quintile

^f^Prior non-breast cancer is a binary variable, with ‘No’ acting as the reference level

The odds of being a non-adherent screener increased with every 5-year increase in age (OR 1.19, 95% CI 1.17, 1.22). Compared to those living in an urban area of over 1,500,000 people, the odds of being a non-screener were significantly lower for those living in smaller urban areas, including communities with a population under 100,000 (OR 0.79, 95% CI 0.71, 0.84), between 100,000 and 499,999 people (OR 0.84, 95% CI 0.78, 0.90), and for those living in a rural area (OR 0.78, 95% CI 0.72, 0.86). An association between neighbourhood income and screening status was also observed. Specifically, the odds of being a non-adherent screener compared to an adherent screener were significantly higher for those in the lowest income category (OR 1.25, 95% CI 1.15, 1.37), but not those in the middle-income category compared to the highest (OR 1.02, 95% CI 0.95, 1.09).

A significant interaction between community population size and income was observed (*p* = 0.04). In a model stratified by community population size, it was found that the effect of income on screening behaviour differed by community population size (Table 3). Specifically, lower income was associated with an increased odds of being a non-screener across all urban areas but was not associated with screening adherence among those living in a rural setting. For urban areas over 1,500,000 people, the odds of being a non-screener were highest for women in the lowest versus highest income category (OR 1.52, 95% CI 1.38, 1.67), and there were lower odds of being a non-screener for women in the middle versus lowest income category (OR 0.75, 95% CI 0.69, 0.81). This pattern was consistent across urban areas of different population sizes (Table 3).

**Table 3:** Multinomial logistic regression measuring the association between age, neighbourhood income, and prior non-breast cancer with the screening behaviours of Ontario women diagnosed with breast cancer from 2010 to 2017, stratified by population size (*n* = 48,184^a^)

Variables^b^	Non-adherent versus adherent screening^c^									
	Community size^d^									
	Urban area over 1,500,000		Urban area 500,00–1,499,999		Urban area 100,000–499,999		Urban area under 100,000		Rural area	
	OR	95% CI	OR	95% CI	OR	95% CI	OR	95% CI	OR	95% CI
Age^e^	1.23	(1.19, 1.27)	1.19	(1.13, 1.26)	1.15	(1.10, 1.20)	1.18	(1.10, 1.27)	1.16	(1.09, 1.24)
Neighbourhood income^f^										
Lowest vs. Highest	1.24	(1.08, 1.42)	1.24	(0.99, 1.55)	1.24	(1.05, 1.48)	1.19	(0.90, 1.58)	1.42	(1.09, 1.85)
Middle vs. Highest	1.07	(0.96, 1.19)	1.02	(0.85, 1.21)	0.97	(0.85, 1.12)	0.83	(0.66, 1.05)	1.14	(0.91, 1.43)
Middle vs. Lowest	0.86	(0.77, 0.97)	0.82	(0.68, 1.00)	0.78	(0.68, 0.90)	0.70	(0.55, 0.88)	0.81	(0.66, 0.99)
Prior non-breast cancer^g^										
Yes vs. No	0.94	(0.80, 1.12)	1.04	(0.80, 1.36)	1.19	(0.99, 1.44)	0.89	(0.64, 1.24)	0.96	(0.77, 1.19)

	Non-Screening versus Adherent Screening^c^									
	Community Size^d^									
	Urban area over 1,500,000		Urban area 500,00–1,499,999		Urban area 100,000–499,999		Urban area under 100,000		Rural area	
	OR	95% CI	OR	95% CI	OR	95% CI	OR	95% CI	OR	95% CI
Age^e^	0.94	(0.92, 0.96)	0.94	(0.91, 0.97)	0.91	(0.89, 0.95)	0.92	(0.88, 0.97)	0.94	(0.90, 0.98)
Neighbourhood income^f^										
Lowest vs. Highest	1.52	(1.38, 1.67)	1.45	(1.24, 1.68)	1.42	(1.23, 1.63)	1.41	(1.14, 1.74)	1.09	(0.90, 1.32)
Middle vs. Highest	1.14	(1.05, 1.23)	0.97	(0.86, 1.09)	1.09	(0.97, 1.22)	1.13	(0.95, 1.34)	1.02	(0.88, 1.19)
Middle vs. Lowest	0.75	(0.69, 0.81)	0.67	(0.59, 0.76)	0.77	(0.69, 0.86)	0.80	(0.68, 0.95)	0.94	(0.81, 1.09)
Prior non-breast cancer^g^										
Yes vs. No	1.04	(0.92, 1.17)	0.92	(0.77, 1.12)	0.91	(0.77, 1.08)	1.04	(0.83, 1.30)	0.96	(0.77, 1.19)

*OR* odds ratio, *CI* confidence interval

^a^Women with ‘Unknown’ Population Size (*N* = 743) are excluded from this stratified analysis

^b^In this model there is no focal exposure. The relationships of all covariates with screening behaviours are explored

^c^Screening behaviour is considered the outcome of interest for this analysis. Adherent screening behaviour is considered the reference level, and the relationships of adherent screening vs non-adherent or non-screening is shown

^d^Urban and rural community size are a categorical variable. Rural areas are defined as surrounding urban areas that have no population centre of their own. Urban areas with over 1,500,000 people act as the reference level for this variable

^e^Age is a continuous variable increasing in units of 5

^f^Neighbourhood income is a categorical variable with three levels. Highest represents the wealthiest neighbourhood income quintile. Middle represents the combined middle three income quintiles. Lowest represents the poorest neighbourhood income quintile

^g^Prior non-breast cancer is a binary variable, with ‘No’ acting as the reference level

A similar pattern was observed for women who were non-adherent screeners compared to adherent screeners. Specifically, lower income was associated with an increased odds of being a non-screener across all populations but was not associated with screening adherence among those in an urban area under 100,000. For rural areas, the odds of being a non-adherent screener were highest for women in the lowest versus highest income category (OR 1.42, 95% CI 1.09, 1.85), and there were lower odds of being a non-adherent screener for women in the middle versus lowest income category (OR 0.81, 95% CI 0.66, 0.99). This pattern was consistent across urban areas of different population sizes except for urban areas under 100,000 (Table 3).

### Screening behaviour and tumour stage at diagnosis

Non-screeners had 9% lower odds of being diagnosed with DCIS compared to adherent screeners (OR 0.91, 95% CI 0.84, 0.98) (Table 4). Conversely, compared to stage I disease, the odds of being diagnosed with stage II (OR 1.91, 95% CI 1.82, 2.01), III (OR 2.96, 95% CI 2.76, 3.17), or IV (OR 8.96, 95% CI 7.94, 10.12) breast cancer were higher for non-screeners compared to adherent screeners and increased with increasing stage.

**Table 4:** Multinomial logistic regression measuring the association between breast cancer screening behaviour, age, neighbourhood population, and prior non-breast cancer with cancer stage in Ontario women diagnosed with breast cancer from 2010 to 2017 (*n* = 48,927)

Adjusted risk factor^a^	Cancer stage^b^											
	In-situ		Non-Staged^c^		II		III		IV		Unknown	
	OR	95% CI	OR	95% CI	OR	95% CI	OR	95% CI	OR	95% CI	OR	95% CI
Screening behaviour^d^												
Non-Adherent vs. Adherent Screening	0.96	(0.88, 1.06)	1.36	(0.90, 2.05)	1.33	(1.24, 1.42)	1.39	(1.26, 1.55)	2.36	(1.97, 2.82)	1.10	(0.98, 1.23)
Non-Screening vs. Adherent Screening	0.91	(0.84, 0.98)	1.34	(0.96, 1.86)	1.91	(1.82, 2.01)	2.96	(2.76, 3.17)	8.96	(7.94, 10.12)	1.57	(1.45, 1.70)
Age^e^	0.90	(0.87, 0.92)	0.97	(0.86, 1.08)	0.89	(0.88, 0.91)	0.85	(0.83, 0.87)	0.96	(0.93, 1.00)	0.90	(0.88, 0.93)
Community size^f^												
Urban 500,000–1,499,999	1.10	(1.01, 1.20)	0.92	(0.61, 1.37)	1.10	(1.03, 1.17)	1.26	(1.15, 1.38)	0.96	(0.83, 1.12)	0.68	(0.60, 0.76)
Urban 100,000–499,999	0.84	(0.77, 0.91)	0.48	(0.31, 0.75)	0.98	(0.92, 1.04)	0.99	(0.91, 1.08)	1.32	(1.17, 1.50)	0.73	(0.66, 0.80)
Urban < 100,000	0.78	(0.69, 0.88)	0.83	(0.50, 1.38)	1.02	(0.94, 1.10)	0.99	(0.88, 1.12)	1.10	(0.92, 1.31)	0.70	(0.61, 0.80)
Rural	0.86	(0.78, 0.96)	0.49	(0.28, 0.86)	1.00	(0.93, 1.07)	1.00	(0.90, 1.12)	1.05	(0.89, 1.24)	0.73	(0.65, 0.82)
Unknown	0.60	(0.44, 0.82)	1.42	(0.57, 3.52)	0.95	(0.79, 1.14)	0.84	(0.63, 1.12)	0.74	(0.47, 1.16)	1.76	(1.41, 2.20)
Prior non-breast cancer^g^												
Yes vs. No	1.05	(0.93, 1.18)	1.01	(0.60, 1.73)	0.91	(0.84, 0.99)	0.88	(0.78, 1.00)	0.77	(0.63, 0.94)	1.28	(1.14, 1.45)

*OR* odds ratio, *95% CI* 95% wald confidence intervals

^a^Model adjusted for age, community size, and presence of a prior non-breast cancer

^b^Cancer stage is a categorical variable representing the outcome of interest for this model. Stage one cancer is used as the reference level for cancer stage

^c^Non-staged cancer represents invasive non-staged breast cancers such as Paget's Disease, Phyllodes tumours, and angiosarcoma of the breast

^d^Screening behaviour is a categorical variable representing the focal exposure of interest in this model. Data on screening episodes come from the OBSP. Compliant screening is the reference level for this variable. Non-adherent screening is when a participant has greater than 3 years between screens for biennial recall and more than 2 years between screens for annual recall. All those who did not have screening records in OBSP were classified as non-screening individuals for this cohort

^e^Age is a continuous variable increasing in units of 5

^f^Urban and rural community size are a categorical variable. Rural areas are defined as surrounding urban areas that have no population centre of their own. Urban areas with over 1,500,000 people act as the reference level for this variable

^g^Prior non-breast cancer is a binary variable, with ‘No’ acting as the reference level

A similar pattern was observed among non-adherent screeners compared to adherent screeners. While non-adherent screeners had 4% non-significantly lower odds of being diagnosed with DCIS compared to adherent screeners (OR 0.96, 95% CI 0.88, 1.06), the odds of being diagnosed with stage II (OR 1.33, 95% CI 1.24, 1.42), III (OR 1.39, 95% CI 1.26, 1.55), or IV (OR 2.36, 95% CI 1.97, 2.82) breast cancer were significantly higher for non-adherent screeners compared to adherent screeners and increased with increasing stage.

## Discussion

Overall, among women aged 51–74 years diagnosed with breast cancer in Ontario between 2010 and 2017, those that were screened according to OBSP guidelines were less likely to be diagnosed with later stage disease. In particular, non-screeners had an almost ninefold higher odds of being diagnosed with stage IV disease. However, as expected, these women were less likely to be diagnosed with DCIS (OR 0.91, 95% CI 0.84, 0.98). In addition, we found that Ontario women residing in urban areas with lower neighbourhood income had higher odds of being a non-screener.

Among women with breast cancer who were eligible to be screened in the OBSP (i.e. women ages 50–74 years), most were adherent screeners (*n* = 26,108, 53.4%), while a minority did not screen at all, or were exclusively screened outside of OBSP (*n* = 16,273, 33.3%). This is consistent with previous research [14]. Notably, very few women (*N* = 6,546, 13.4%) engaged in non-adherent screening (i.e. had at least one screening mammogram but did not follow OBSP guidelines). This suggests that individuals who get screened tend to participate fully in the programme, following OBSP guidelines. Focus on efforts to increase screening initiation in women living in larger urban areas with lower neighbourhood income will increase screening rates among screen-eligible women, at least among those later diagnosed with breast cancer. Further, increasing screening rates is likely to reduce the number of late-stage cancers diagnosed, improving cancer outcomes.

Prior work from the OBSP has shown that about 83% of OBSP women who initiated screening returned for a subsequent screen, and that this proportion consistently increased from 1992 to 2001 [14]. However, more recent trends suggest that screening retention has actually decreased in Ontario from 83% in 2012 to 77% in 2018 [3]. In particular, it has been shown that the odds of returning for a second screen are highest for those living in rural compared to urban areas [15]. However, when compared to the broader literature, the impact of rural versus urban living on screening behaviour is mixed. Some studies based in the United States have found that access to breast screening is more available in urban centres. This is reflected in the higher screening rates for women living in urban versus rural areas [7, 16, 17]. Other studies, in Australia and Croatia (both with publicly funded screening), have shown similar screening rates in women living in rural and urban settings [18, 19]. Overall, the literature suggests that access to screening services does tend to be lower for women living in rural areas [18, 20]. On the contrary, in our study, we found that women living in rural areas were less likely to be non-screeners. This suggests that having an organized, province-wide, publicly funded screening programme mitigates some of the rural–urban disparities in screening rates observed in other jurisdictions.

Data have also shown that those in the lowest neighbourhood income category tend to have higher odds of not being screened compared to those in the middle- or highest neighbourhood income categories [8, 21, 22]. Consistent with the evidence, in our study, individuals in lower- and middle-income neighbourhoods were more like to be non-screeners when compared to those in the highest income quintile. This is despite the fact that OBSP screening is publicly available, without the need for referral from primary care, and has no associated cost for the patient [23]. Prior research of low-income African American women in the United States has shown that mistrust of the medical system, inadequate education about screening, and the presence of barriers (e.g. lack of childcare and transportation) may limit the ability of some individuals to attend screening [24, 25]. Work is needed to determine if similar barriers to screening exist in Ontario, preventing low- and middle-income women from attaining the same degree of screening as their higher-income counterparts.

Notably, while we did observe differences in screening rates with neighbourhood income, when comparing non-screeners to screeners, this effect was limited to women living in an urban setting, with no observed differences in screening behaviour based on neighbourhood income for individuals living in a rural area. When comparing non-screeners to screeners in urban areas, differences in screening behaviour were observed for individuals with low, middle, and high neighbourhood income. Here, the odds of being a non-screener were highest for individuals in the lowest income category living in large urban centres (OR 1.52, 95% CI 1.38, 1.67 compared to high-income individuals). This suggests that screening behaviour differences associated with the effects of income are most persistent for those living in urban environments and not in rural areas. Similar research into the interaction of income and community size on breast screening has been performed in low-income countries [26]. Here, it was found that both urban and rural residing women residing in lower-income neighbourhoods had significantly lower odds of mammography attendance relative to women in higher-income neighbourhoods; however, the effect size for rural residing women was smaller in comparison with urban residing women [26]. Further research into breast cancer screening behaviour should focus on what specific barriers to screening exist for women living in low-income, urban neighbourhoods that do not exist for women residing in low-income rural areas.

The effectiveness of breast cancer screening programmes in reducing the incidence of advanced stage breast cancer has been shown in multiple studies [4, 5, 27–29]. Consistent with these findings, the results from this analysis show a clear gradient of an increasing odds of stage II (OR 1.91, 95% CI 1.82, 2.01), III (OR 2.96, 95% CI 2.76, 3.17), and IV (OR 8.96, 95% CI 7.94, 10.12) cancers in non-screeners. A similar pattern was observed for non-adherent screeners, albeit with attenuated effect sizes (Table 4). These results suggest that, while any screening is beneficial, regular breast cancer screening according to OBSP guidelines, is most effective in reducing later stage cancer diagnoses. While performance measures of the OBSP have been analysed [14, 30, 31], to our knowledge, this is the first analysis to look at differences in cancer stage for breast cancer patients who did or did not participate in the OBSP during this time-period. These results highlight the effectiveness of breast screening in Ontario in achieving the goal of reducing the incidence of later stage disease.

Rates of DCIS diagnosis are known to be higher in a screening population [32]. Accordingly, in this study we found non-screeners to be less likely to be diagnosed with DCIS compared to adherent screeners (OR 0.91, 95% CI 0.84, 0.98). It was expected that more cases of DCIS would be found in adherent screeners (11.7%) compared to non-adherent (9.8%) and non-screeners (7.1%).

This study has numerous strengths, including the use of a large population-based cohort of women diagnosed with breast cancer identified through a provincial cancer registry. This allowed for robust comparisons between adherent screeners and non-screeners. The OCR also includes detailed information on tumour characteristics (e.g. stage, ER-status) that can be linked to demographic characteristics of the women within this population.

Limitations of this study include a lack of information on personal income levels, race/ethnicity, and immigration status of women diagnosed with breast cancer in Ontario. The absence of these key demographic variables limited our ability to examine their impact on breast cancer screening behaviours and therefore limited the conclusions we could draw from this current analysis. Further, the study population only included women who were diagnosed with breast cancer, so when examining demographic characteristics associated with breast cancer screening behaviour, these relationships may only exist among women diagnosed with breast cancer. However, screening behaviours were captured through OBSP records and only included screens conducted prior to diagnosis, and all women known to be at high risk of breast cancer (i.e. screened as part of the High Risk OBSP) were excluded from the analysis. Because the coverage of the OCR and the OBSP is province-wide, it is expected that the screening behaviours in this study population reflect those of the broader population. Further evidence of this is seen in the similar screening rates (66.7%) in the study population, as compared to the general population of Ontario in 2018 (66.0%) [3]. Another limitation is that eligible women who exclusively screened outside OBSP would have been classified as non-screeners in this sample since only OBSP screening data were available. However, it would be expected that screening adherent women misclassified as non-screening would attenuate the associations seen with cancer stage at diagnosis.

This research highlights the important differences between women who are screened according to the guidelines of a province-wide, publicly funded screening programme, and those who are screened, but do not meet these guidelines or do not get screened at all. Women who underwent any screening were less likely to have been diagnosed with late-stage breast cancer; however, comparing screen adherent women to non-screening women showed the strongest association. Furthermore, being younger, urban residing, or having a lower neighbourhood income were all associated with a greater likelihood of not undergoing any breast cancer screening. Notably, few women were non-adherent screeners, suggesting that once women initiate screening, most tend to follow the guidelines set out by OBSP. This highlights the need for focused interventions aimed at increasing screening initiation among urban residing, low-income women, to increase screening rates and ensure that more breast cancers are detected before they progress to more advanced and serious stages.

## Data Availability

The data that support the findings of this study are available from Ontario Health (Cancer Care Ontario), a prescribed entity under Section 45 of the Personal Health Information Protection Act. Data sharing regulations prevent these data from being made available publicly due to the personal health information in the datasets. Data are, however, available from the authors upon reasonable request and with permission of Ontario Health (Cancer Care Ontario).
